# Associations between interleukin and interleukin receptor gene polymorphisms and risk of gout

**DOI:** 10.1038/srep13887

**Published:** 2015-09-24

**Authors:** Shiguo Liu, Zheng Zhou, Can Wang, Mingzhen Guo, Nan Chu, Changgui Li

**Affiliations:** 1 Prenatal Diagnosis Center, the Affiliated Hospital of Qingdao University, Qingdao, 266003, China; 2 Institute of Clinical Research, the Affiliated Hospital of Qingdao University, Qingdao, 266003, China; 3 Shandong Provincial Key Laboratory of Metabolic Disease, the Affiliated Hospital of Qingdao University, Qingdao, 266003, China; 4 Qingdao HaiCi hospital, Qingdao, 266003, China

## Abstract

Gout is a self-limiting, auto-inflammatory arthritis induced by the deposition of monosodium urate crystals in the synovial fluid and periarticular tissues. The aim of this study was to investigate the associations between genetic variants in the interleukin (IL) and interleukin receptor (ILR) genes *IL-33*, *IL-1RL1*, *IL-23R*, and signal transducer and activator of transcription 4 (*STAT4*) and susceptibility to gout in Chinese Han male individuals. The genetic distributions of rs3939286 in *IL-33*, rs13015714 in *IL-1RL1*, rs10889677 in *IL-23R*, and rs7574865 in *STAT4* were detected in 1100 men with gout and 1227 ethnically matched controls, using Taqman allelic discrimination real-time polymerase chain reaction (PCR). Differences in these polymorphisms between the groups were investigated using χ^2^ tests. The genotype-phenotype relationship among gout patients was tested by analysis of variance. There was a significant difference in genotypic frequencies of IL-23R rs10889677 between gout patients and controls (χ^2^ = 81.386, P < 0.001). However, there were no significant differences in distributions of the other polymorphisms between the groups. Our results revealed that the rs10889677 variant in *IL-23R* may be involved in the development of gout in Chinese Han male individuals. However, further studies in other ethnic groups are needed to confirm these results.

Gout is the most common inflammatory arthritis and is characterized by elevated serum urate levels and precipitation of monosodium urate crystals (MSU) in the joints, resulting in recurrent episodes of acute pain. The incidence of gout is increasing globally in line with an aging population according to the Global Burden of Disease studies^1^. However, the prevalence of gout varies among geographical areas and racial groups^2^, with a prevalence of 2.49% in the UK in 2012, compared with 6.24% in Taiwan in 2010^3^,^4^. Gout is a polygenic hereditary disease and previous studies have focused on genetic variants related to key regulators of uric acid homeostasis, such as *URAT1*, *ABCG2*, and *SLC2A9*^5^,^6^,^7^. However, in a previous epidemiological study, we found that only about 10% of hyperuricemia patients finally developed gout^8^, indicating that candidate genes related to uric acid metabolism are unable to provide a full explanation for the pathogenesis of gout. Increasing numbers of studies have therefore concentrated on variations in genes related to inflammatory mediators in gout.

The deposition of MSU caused by increased uric acid levels in the blood, together with several risk factors, can active the NLRP3 (NACHT, LRR, and PYD domain-containing protein 3) inflammasome, resulting in caspase-1 activation and secretion of functional interleukin (IL)-1β and IL-18, accompanied by the release of other pro-inflammatory cytokines such as IL-6, IL-8, and tumor necrosis factor (TNF)-α^9^,^10^. These cytokines can finally lead to amplification of the inflammatory response, with neutrophil infiltration into the joints and periarticular tissues. IL-33 is an important member of the IL-1 family and a pro-inflammatory cytokine that enhances T-helper cell immune responses by binding to its receptor, IL-1 receptor-like 1 (IL-1RL1) and co-receptor, IL-1 receptor accessory protein (IL-1RAcP)^11^,^12^. The IL-33/IL-1RL1 axis plays a critical role in several autoimmune and inflammatory disorders. IL-23 is a member of the IL-12 family and also a pro-inflammatory cytokine, comprising a p19 subunit and a p40 subunit of IL-12B. After binding to the IL-23 receptor (IL-23R), IL-23 stimulates the secretion of a variety of inflammatory factors such as IL-1, IL-6, IL-8, and TNF-α by activated CD4 + Th17 cells, which in turn drive gouty inflammation^13^. In addition, signal transducer and activator of transcription 4 (STAT4), encoded by *STAT4* mapped to chromosome 2q33, regulates the IL-23-related inflammatory response by transmitting signals in response to several cytokines such as IL-23 and IL-12^14^. STAT4 is also essential for the expansion of Th17 cells activated by IL-23, which contributes to the development of many autoimmune diseases^15^. The production and function of cytokines may be affected by polymorphisms in the functional regions of their genes, suggesting that *IL-33*, *IL-1RL1*, *IL-23R*, and *STAT4* may be candidate genes for the inflammatory pathogenesis of gout. The current study therefore aimed to investigate the associations between the IL-33 rs3939286 A/G, IL-1RL1 rs13015714 G/T, IL-23R rs10889677 A/C, and STAT4 rs7574865 G/T single nucleotide polymorphisms (SNPs) and the risk of gout.

## Results

### Demographic and clinical characteristics

The clinical characteristics of all subjects are summarized in Table 1. The mean ages ( ± SD) of the two groups were 50.98 ± 13.92 and 59.72 ± 14.19 years, respectively. Compared with controls, gout patients had higher diastolic pressure (85.01 ± 11.71 vs. 88.07 ± 12.46 mmHg, P < 0.001), higher uric serum acid levels (313.87 ± 59.31 vs. 473.06 ± 122.44 μmol/L, P < 0.001), higher serum triglyceride (TG) levels (1.68 ± 4.44 vs. 2.36 ± 1.75 mmol/L, P < 0.001), higher total cholesterol (TC) levels (5.33 ± 1.07 vs. 5.47 ± 1.35 mmol/L, P = 0.006), and higher serum creatinine levels (80.80 ± 17.52 vs. 88.80 ± 33.66 μmol/L, P < 0.001). However, other parameters including body mass index (BMI), systolic pressure, and blood glucose levels were similar in both groups (P = 0.967, P = 0.098, and P = 0.964, respectively; Table 1).

### Genetic analysis

The distributions of the four SNPs among the controls were in Hardy–Weinberg equilibrium (for rs3939286, χ^2^ = 2.16, P = 0.142; for rs13015714, χ^2^ = 2.28, P = 0.131; for rs10889677, χ^2^ = 0.973, P = 0.324; for rs7574865, χ^2^ = 0.310, P = 0.578). The results for the whole study population are shown in Tables 2 and 3. We detected a significant difference in genotype frequency of IL-23R rs10889677 (χ^2^ = 81.39, P < 0.001; Table 3) between gout patients and controls. The frequency of the C allele was higher in gout patients compared with controls, but the difference was not significant (P = 0.059, OR = 1.137, 95% CI 0.995–1.298). When the samples were subdivided into AA + AC/CC groups or AA/AC + CC groups, there was a significant difference between cases and controls in terms of AA + AC and CC groups (P < 0.001, OR = 17.589, 95% CI 7.124–43.431). However, no significant differences in genotype or allele frequencies between the groups were identified for the remaining SNPs (for rs3939286, P = 0.478 by genotype, χ^2^ = 1.46, P = 0.309 by allele; for rs13015714, χ^2^ = 2.35, P = 0.770 by genotype, χ^2^ = 0.01, P = 0.228 by allele; for rs7574865, χ^2^ = 0.52, P = 0.905 by genotype, χ^2^ = 0.001, P = 0.972 by allele; Table 2). To confirm the accuracy of the genotyping results, 50 samples including cases and controls were selected randomly for sequencing validation, and the results were consistent.

### Genotype-phenotype analysis of the rs10889677 polymorphism

As shown in Table 4, a detailed genotype-phenotype analysis of IL-23R rs10889677 was conducted among gout patients in relation to clinical factors including demographic characteristics and serum biochemical parameters, as well as tophi and previous medical history (hypertension, diabetes mellitus, and obesity). When gout patients were divided into AA, AC, and CC groups, there were significant differences in hypertension history between the three groups (P = 0.020) and between the AA and AC + CC groups (P = 0.009). However, there were no significant differences between the three groups in any other parameters, including BMI, systolic pressure, diastolic pressure, other serum biochemical indexes, tophi, diabetes mellitus, or obesity. The results were as the same when the patients were subdivided into AA + AC/CC groups or AA/AC + CC groups (all P > 0.05; Table 4).

## Discussion

Gouty inflammation is a paradigm of innate immunity and the IL-1β/IL-1R pathway plays a key role in acute gout attack^16^. Phagocytosis of MSU by macrophages can activate the NLRP3 inflammasome, which is considered to play a critical role in gouty arthritis. Caspase-1 is in turn activated by the NLRP3 inflammasome and can lead to the conversion of pre-IL-1β and pre-IL-18 to mature IL-1β and IL-18 and secretion of the latter. Binding of IL-1β to the IL-1β receptor in endothelial cells and resident macrophages, together with other pro-inflammatory cytokines and chemokines such as IL-6, IL-8, and TNF-α, can finally lead to gouty inflammation^17^.

Genetic factors are known to play key roles in the pathogenesis of gout, and genetic variants in genes for cytokines involved in gouty inflammatory pathways, such as IL-1β, IL-1R, IL-6, IL-8, and IL-18, have been associated with susceptibility to gout^17^. We previously suggested that the IL-8 251T/A and IL-12B 1188A/C polymorphisms may be relevant host susceptibility factors for the development of gout^18^. In addition, a case-control study in 400 gout patients and 582 gout-free controls revealed that the IL-23R rs7517847 G/T polymorphism might be associated with gout in Chinese Han males^19^. Moreover, the analysis of linkage disequilibrium between rs7517847 and rs10889677 in *IL-23R* was done further, and the D’ value was 0.653, while the r^2^ value was 0.157, which indicated that there was no linkage disequilibrium between these two SNPs. Sites rs10889677 and rs7517847 of *IL-23R* were independent to each other and this may demonstrated *IL-23R* as an important candidate gene for gout susceptibility. However, Tsai *et al.*^20^ found no significant association between four polymorphisms in the IL-6 gene promoter, including -597G/A, -572C/G, -373A/T, and -174G/C and gout.

As an IL-1 family member, IL-33 is synthesized as a 30-kDa peptide and then cleaved by caspase-1 to form an active 18-kDa mature peptide, which is widely expressed in tissues, specifically in epithelial linings and smooth muscle cells^21^. Through binding to the IL-33R encoded by *IL-1RL1*, IL-33 can activate mast cells and Th2 cells as well as Th17 cells, leading to the production of pro-inflammatory cytokines and chemokines^21^. IL-23/IL-23R represents a novel pathway involved in inflammatory responses. The effect of IL-23 depends on its binding to IL-23R, a heterodimer of the IL-12RB1 subunit^22^, which was identified as an additional candidate gene in some autoimmune and inflammatory diseases such as inflammatory bowel disease, psoriasis, Graves’ ophthalmopathy, ankylosing spondylitis, and multiple sclerosis^23^,^24^. A genome-wide association study in Japanese subjects reported that the rs12119179 variant in *IL-23R* was associated with Behcet’s disease^25^,^26^. As noted above, IL-23 may be associated with the production of IL-17A, IL-17F, IL-6, and TNF-α by active Th17 cells, which are important effector cytokines during gouty inflammation. In return, the engagement of IL-17 and/or IL-17F with endothelial cells promotes the expression of IL-1, IL-6, IL-8, TNF-α, and ICAM-1, which are critical factors driving the inflammatory response^13^. Moreover, the ability of IL-23 to induce IL-17A, IL-17F, and other pro-inflammatory factors appears important for neutrophil recruitment^27^. STAT4 transmits signals induced by several cytokines such as IL-12 and IL-23, which are key cytokines in the development of autoimmune diseases^14^. Furthermore, STAT4 plays a key role in IL-12-induced T-cell differentiation into Th1 cells^14^ and in IL-23-mediated production of IL-17 by activated Th17 cells^15^,^28^. The functions of these inflammatory cytokines suggest that they may play important roles in the inflammatory pathogenesis of gout. We therefore propose *IL-33*, *IL-1RL1*, *IL-23R*, and *STAT4* as potential candidate susceptibility genes for gout.

To the best of our knowledge, this is the first study to investigate the association between these SNPs in IL-1-related cytokine genes and the risk of gout. We found that IL-23R rs10889677 was significantly associated with susceptibility to gout in Chinese Han male individuals. The genotypic frequency differed significantly between gout patients and controls, and patients with the AC or CC genotype were more likely to develop gout than patients with the AA genotype. It found that significant differences exist in patients with hypertension history between the AA and AC + CC groups and between the three groups when doing an analysis on the genotype-phenotype of IL-23R rs10889677, which showed C allele may be a risk allele for gout patients to develop hypertension, however, it may need further research as C allele frequency of rs10889677 is smaller than A allele. rs10889677 is located in the 3′-UTR of *IL-23R*, suggesting that it might regulate the expression and function of IL23R mRNA. The other three tested SNPs showed no significant association with gout risk. However, Carriere *et al.* and Oboki *et al.* reported that IL-33 rs3939286 and IL-1RL1 rs13015714 were associated with other autoimmune diseases such as Crohn’s disease, rheumatoid arthritis (RA), asthma, and Alzheimer’s disease^29^,^30^. Hayakawa *et al.* also found that blocking IL-1RL1 receptor signaling could prevent arthritis development and airway inflammation^31^,^32^. A relevant study in western Algeria indicated that the STAT4 rs7574865 polymorphism was clearly associated with the risk of RA in an Algerian population^33^, while a meta-analysis by Jiang *et al.* also indicated a significant association between the rs7574865 polymorphism and a decreased RA risk in an Asian population^34^. In addition, Liu *et al.* found that rs7574865 in *STAT4* was significantly associated with increased susceptibility to and severity of ankylosing spondylitis in a Chinese Han Population.

There were some limitations to this study. First, all the subjects were Chinese Han individuals from Shandong Province, and further studies including participants from other nationalities and regions are therefore needed to determine the general applicability of the results, given the important roles of both genetic and environmental factors in the development of gout. Furthermore, other important SNPs exist on these genes, and the contributions of these to gout susceptibility should also be investigated. However, despite these limitations, our study demonstrated an association between *IL-23R* and susceptibility to gout in Chinese Han male individuals.

## Methods

### Populations and clinical data

A total of 1100 men with gout who visited the Department of Gout, the Affiliated Hospital of Qingdao University, China between January 2009 and December 2014 were recruited to this study. Gout diagnosis followed the criteria published by the American College of Rheumatology in 1997^35^. No patients had any history of cancer, hematopathy, nephropathy, or other autoimmune diseases. Some metabolic diseases such as hypertension, obesity, diabetes and so on were not excluded from gout patients because there were no researches have indicated relationships between the four SNPs and these diseases. A further 1227 gout-free male controls were enrolled. All the participants were Chinese Han men. Blood samples were collected into anticoagulant-coated tubes and transferred immediately to the laboratory for DNA extraction and genetic analysis. Demographic and laboratory parameters, and medical histories were recorded by experienced endocrinologist physicians, including serum uric acid, glucose, TG, TC and creatinine levels, blood pressure, and BMI, as well as age of onset, tophi, and medical history among gout patients. This study was approved by the Ethics Committee of the Affiliated Hospital, Qingdao University and conducted in accordance with the ethical guidelines of the 1975 Declaration of Helsinki.

### DNA extraction and genetic analysis

After obtaining informed consent, genomic DNA was extracted from peripheral blood samples from gout patients and controls using genomic DNA Extraction kits (Qiagen, Hilden, Germany) in accordance with the manufacturer’s instructions. All four SNPs (rs3939286, rs13015714, rs10889677, rs7574865) were genotyped by CFX96^TM^ real-time polymerase chain reaction (PCR) (Bio-Rad, California, USA) using the TaqMan probe method. The TaqMan probes and primers were designed and synthesized by Applied Biosystems of Life Technologies (New York, USA). The sequences of the forward and reverse primers were: rs3939286, 5′-TCCACATCCCCATGGTTTGTTGTTG-3′ and 5′-TGCTTGTAGTGGGTTGTTGTTATCT-3′, respectively; rs13015714, 5′-CGGCTATGGGTTTCCCTTTTCCTTT-3′ and 5′-GTTAAATAACAGTTCTGCCACAAAA-3′, respectively; rs10889677, 5′-TTTAATTTTAGCCATTCTTCTGCCT-3′ and 5′-ATTTCTTAAAATTAGAGAATTAAGG-3′, respectively; rs7574865, 5′-TATGAAAAGTTGGTGACCAAAATGT-3′ and 5′-AATAGTGGTTATCTTATTTCAGTGG-3′, respectively. The amplifications were carried out by C1000TM thermal cycler in a 25-μL total reaction volume containing 12.5 μL 2× PCR Master Mix, 1.25 μL 20× SNP Genotyping Assay, 11.25 μL DNA sample, and DNase-free water. The PCR conditions were as follows: initial denaturation at 95 °C for 3 min, followed by 45 cycles of denaturation at 95 °C for 15 s and 60 ˚C 1 min. Genotypes of all the samples were finally analyzed using Bio-Rad CFX manager 3.0 software.

### Statistical analysis

Statistical analyses were carried out using the Statistical Package for Social Sciences version 22.0 (SPSS Inc., Chicago, IL, USA). The Hardy–Weinberg equilibrium of the control group was tested using the goodness-of-fit χ^2^ test. Differences in genotypes and alleles between cases and controls were analyzed using the Pearson’s χ^2^ test (or Fisher’s exact test if the expected values were < 5), and P-values < 0.013 were considered significant when Bonferroni’s correction was made. Differences in demographic and clinical indexes between the two groups were compared using the Student’s t-test. Differences between genotyping and clinical characteristics in gout patients, including clinical and biochemical parameters, tophi, and past medical history were assessed by analysis of variance. The strength of the relationships in allelic distributions between gout patients and controls was assessed by OR and 95% CI. All statistical tests were two-sided with a significance level of 0.05, and differences were deemed to be significant at P < 0.01.

## Additional Information

**How to cite this article**: Liu, S. *et al.* Associations between interleukin and interleukin receptor gene polymorphisms and risk of gout. *Sci. Rep.* **5**, 13887; doi: 10.1038/srep13887 (2015).

## Figures and Tables

**Table 1:** Demographic and clinical characteristics of cases and controls.

characteristic	Gases (n = 1100)	Controls(n = 1227)	t	P
Age (years)	50.98 ± 13.92	59.72 ± 14.19	−14.59	<0.001
BMI (kg/m^2^)	27.00 ± 3.49	26.91 ± 70.00	0.04	0.967
Systolic pressure (mmHg)	135.47 ± 19.40	134.06 ± 19.61	1.66	0.098
Diastolic pressure (mmHg)	88.07 ± 12.46	85.01 ± 11.71	5.80	<0.001
Blood glucose (mmol/L)	6.16 ± 2.14	6.15 ± 7.02	0.05	0.964
Uric acid (μmol/L)	473.06 ± 122.44	313.87 ± 59.31	40.38	<0.001
Triglycerides (mmol/L)	2.36 ± 1.75	1.68 ± 4.44	0.49	< 0.001
Total cholesterol (mmol/L)	5.47 ± 1.35	5.33 ± 1.07	2.78	0.006
Creatinine (μmol/L)	88.80 ± 33.66	80.80 ± 17.52	5.25	<0.001

BMI: Body Mass Index.

*P-value of Fisher’s exact test.

**Table 2:** The genotypic and allelic frequencies of rs3939286, rs13015714 and rs7574865 between cases and controls.

	Cases(n = 1100)	Controls(n = 1227)	χ^2^	P-value#	OR(95%CI)
rs3939286					
Genotypes					
AA	1	1			
AG	101	132		0.478*	
GG	998	1094			
Alleles					
A	103	134			
G	2097	2320	1.46	0.228	0.850(0.653–1.107)
rs13015714					
Genotypes					
GG	329	350			
GT	535	635	2.35	0.309	
TT	236	242			
Alleles					
G	1193	1335			
T	1007	1119	0.01	0.905	0.993(0.885–1.115)
rs7574865					
Genotypes					
GG	487	533			
GT	483	558	0.52	0.770	
TT	130	136			
Alleles					
G	1457	1624			
T	143	830	0.001	0.972	1.002(0.887–1.132)

^*^P-value of Fisher’s exact test.

^#^P-value < 0.013 was considered significant after Bonferroni’s correction.

**Table 3:** The genotypic and allelic frequencies of 10889677 between cases and controls.

	Cases(n = 1100)	Controls(n = 1270)	χ^2^	P-value#	OR(95%CI)
Genotypes					
AA	578	672			
AC	517	463	81.39	<0.001	
CC	5	92			
AA + AC	1095	1135			
CC	5	92	71.31	<0.001	17.589(7.124–43.431)
AA	578	672			
AC + CC	522	555	1.15	0.283	0.914(0.777–1.077)
Alleles					
A	1673	1807			
C	527	647	3.57	0.059	1.137(0.995–1.298)

^#^P-value < 0.013 was considered significant after Bonferroni’s correction.

**Table 4:** Association between genotypes of rs10889677 and clinical characteristics among gout patients.

rs10889677 n	(1) AA n	(2) AC n	(3) CC n	(1) vs.(2) vs.(3)	(1) + (2)vs. (3)	(1) vs.(2) + (3)
1100	578	517	5	P	P	P
Clinical and serum biochemical indexes (mean ± SD)						
Age of onset	44.57 ± 13.51	45.69 ± 13.53	49.80 ± 13.99	0.309	0.663	0.169
BMI (kg/m2)	26.35 ± 5.65	25.80 ± 6.30	27.06 ± 3.29	0.309	0.534	0.144
Systolic pressure (mmHg)	135.87 ± 19.50	134.90 ± 19.12	148.50 ± 34.30	0.297	0.281	0.488
Diastolic pressure (mmHg)	88.09 ± 11.94	87.98 ± 12.92	94.25 ± 23.21	0.604	0.435	0.948
Blood glucose (mmol/L)	6.13 ± 2.45	6.19 ± 1.73	5.61 ± 0.40	0.783	0.424	0.719
Uric acid (μmol/L)	470.65 ± 120.93	476.00 ± 123.60	453.33 ± 180.94	0.593	0.795	0.490
Triglycerides (mmol/L)	2.33 ± 1.58	2.40 ± 1.94	1.74 ± 1.41	0.393	0.351	0.551
Total cholesterol (mmol/L)	5.16 ± 1.21	5.24 ± 1.19	5.58 ± 0.16	0.345	0.776	0.226
Creatinine (μmol/L)	86.23 ± 30.19	89.39 ± 39.93	89.20 ± 8.61	0.724	0.939	0.149
Clinical data (n(%))						
Tophi	102/578	113/517	1/5	0.169*	0.999*	0.104
Hypertension^a^	196/578	214/517	3/5	0.020*	0.370	0.009
Diabetes^b^	45/578	39/517	0/5	0.939*	0.999*	0.845
Obesity^c^	213/578	165/517	1/5	0.187*	0.665	0.078

BMI: body mass index.

^*^P-value of Fisher’s exact test.

^a^Hypertension: systolic pressure ≥140 mmHg or diastolic pressure ≥90 mmHg or having a previous history of hypertension and receiving anti-hypertensive medication.

^b^Diabetes: fasting blood glucose ≥7.0 mmol/L (126 mg/dL) or random blood glucose ≥11.1 mmol/L (200 mg/dL) or under treatment.

^c^Obesity: BMI ≥ 28 kg/m^2^.
